# Tumor-Initiating Cells Are Enriched in CD44^hi^ Population in Murine Salivary Gland Tumor

**DOI:** 10.1371/journal.pone.0023282

**Published:** 2011-08-16

**Authors:** Shukun Shen, Wenjun Yang, Zhugang Wang, Xia Lei, Liqun Xu, Yang Wang, Lizhen Wang, Lei Huang, Zhiwei Yu, Xinhong Zhang, Jiang Li, Yan Chen, Xiaoping Zhao, Xuelai Yin, Chenping Zhang

**Affiliations:** 1 Department of Oral and Maxillofacial Surgery, College of Stomatology, Ninth People's Hospital, Shanghai Jiao Tong University School of Medicine, Shanghai, People's Republic of China; 2 Laboratory of Oral Tumor and Oral Biology, Shanghai Key Laboratory of Stomatology and Shanghai Research Institute of Stomatology, Shanghai, People's Republic of China; 3 Model Organism Division, Department of Medical Genetics, Shanghai Jiao Tong University School of Medicine, Shanghai, People's Republic of China; 4 Department of Oral Pathology, College of Stomatology, Ninth People's Hospital, Shanghai Jiao Tong University School of Medicine, Shanghai, People's Republic of China; University of Southern California, United States of America

## Abstract

Tumor-initiating cells (T-ICs) discovered in various tumors have been widely reported. However, T-IC populations in salivary gland tumors have yet to be elucidated. Using the established Pleomorphic Adenoma Gene-1 (Plag1) transgenic mouse model of a salivary gland tumor, we identified CD44^high^ (CD44^hi^) tumor cells, characterized by high levels of CD44 cell surface expression, as the T-ICs for pleomorphic adenomas. These CD44^hi^ tumor cells incorporated 5-bromo-2-deoxyuridine (BrdU), at a lower rate than their CD44^negative^ (CD44^neg^) counterparts, and also retained BrdU for a long period of time. Cell surface maker analysis revealed that 25% of the CD44^hi^ tumor cells co-express other cancer stem cell markers such as CD133 and CD117. As few as 500 CD44^hi^ tumor cells were sufficient to initiate pleomorphic adenomas in one third of the wildtype mice, whereas more than 1×10^4^ CD44^neg^ cells were needed for the same purpose. In NIH 3T3 cells, Plag1 was capable of activating the gene transcription of Egr1, a known upregulator for CD44. Furthermore, deletion of sequence 81–96 in the Egr1 promoter region abolished the effect of Plag1 on Egr1 upregulation. Our results establish the existence of T-ICs in murine salivary gland tumors, and suggest a potential molecular mechanism for CD44 upregulation.

## Introduction

Tumors comprise of a heterogeneous population of cells with varying morphologies and functions. It has been proposed that tumors may not be mere monoclonal expansions of cells. Instead, they may be sustained by a specialized type of tumor-initiating cells (T-ICs), also known as cancer stem cells, which are capable of self-renewal and aberrant differentiation [1]. Many tumors have a hierarchical organization of T-ICs, rapidly dividing cells and differentiated tumor cells. These T-ICs are not only a renewable source of tumor cells, but are also a source of tumor resistance, leading to tumor progression, metastasis and recurrence [2]–[4]. Hence, identifying these cells and their respective cell surface markers are crucial to the understanding of the mechanisms that govern cancers, thereby leading to the development of better and more strategic oncological therapies.

Early experimental evidence for T-ICs came from human acute myeloid leukemia studies. Human CD34^+^CD38^−^ acute myeloid leukemia cells were capable of initiating leukemia when transplanted in non-obese diabetic mice with severe combined immunodeficiency (NOD/SCID mice) [5]. More recently, T-ICs were identified and isolated from epithelial tumors such as breast tumors [6]–[8], glioblastomas [9]–[10] and colorectal carcinomas [11], where each of these tissue-specific T-ICs displayed distinct cell surface markers. For instance, those discovered in human breast tumors had a subpopulation of CD44^+^CD24^−/low^ tumor cells, capable of initiating and sustaining tumor growth after they were xenografted into NOD/SCID mice. These CD44^+^CD24^−/low^ cells contained mixed populations of epithelial tumor cells, and often phenocopied the histological heterogeneity of their parent tumors [8]. Another example of T-ICs includes the CD133^+^ tumor cells discovered in glioblastoma multiforme and medulloblastomas of the human brain, where CD133^+^ subpopulations accounted for 5–30% of the total number of tumor cells. These cells were found to be tumorigenic and could reproduce the phenotypic diversity and differentiation pattern of the parent tumors when injected intracerebrally [12]. In human colorectal cancer, the ability to engraft *in vivo* in NOD/SCID mice is restricted to EpCAM^high/^CD44^+^ tumor cells. Tumors originated from EpCAM^high^/CD44^+^ cells also maintained a differentiated phenotype and reproduced the phenotypic heterogeneity of their parental lesions [13].

Pleomorphic adenomas are benign tumors found in salivary glands. They are the most common of all salivary gland tumors, and can account for up to 80% of all parotid neoplasms. While most human pleomorphic adenomas are benign and can be easily treated via various forms of curative resections, some of them may recur decades after removing the primary tumor. An even smaller percentage of them can become malignant (carcinoma ex-pleomorphic adenoma) with metastasis to distant and remote sites [14]–[15]. Investigations on the tumorigenesis of pleomorphic adenomas revealed that the oncogenic activation of Pleomorphic Adenoma Gene 1 (Plag1) played a pivotal role in their development within salivary glands. Various studies including ours verified that Plag1 transgenic mice spontaneously developed salivary gland tumors with similar histopathological features to human pleomorphic adenomas [16]–[18]. Using this model, we sought to establish the presence of T-ICs and identify the corresponding cell surface markers for T-IC isolation. In this study, we provided evidence that CD44^hi^ cells function as the T-ICs in pleomorphic adenomas.

## Results

### CD44 protein expression in normal salivary gland or salivary gland tumor cells

**Figure 1:**
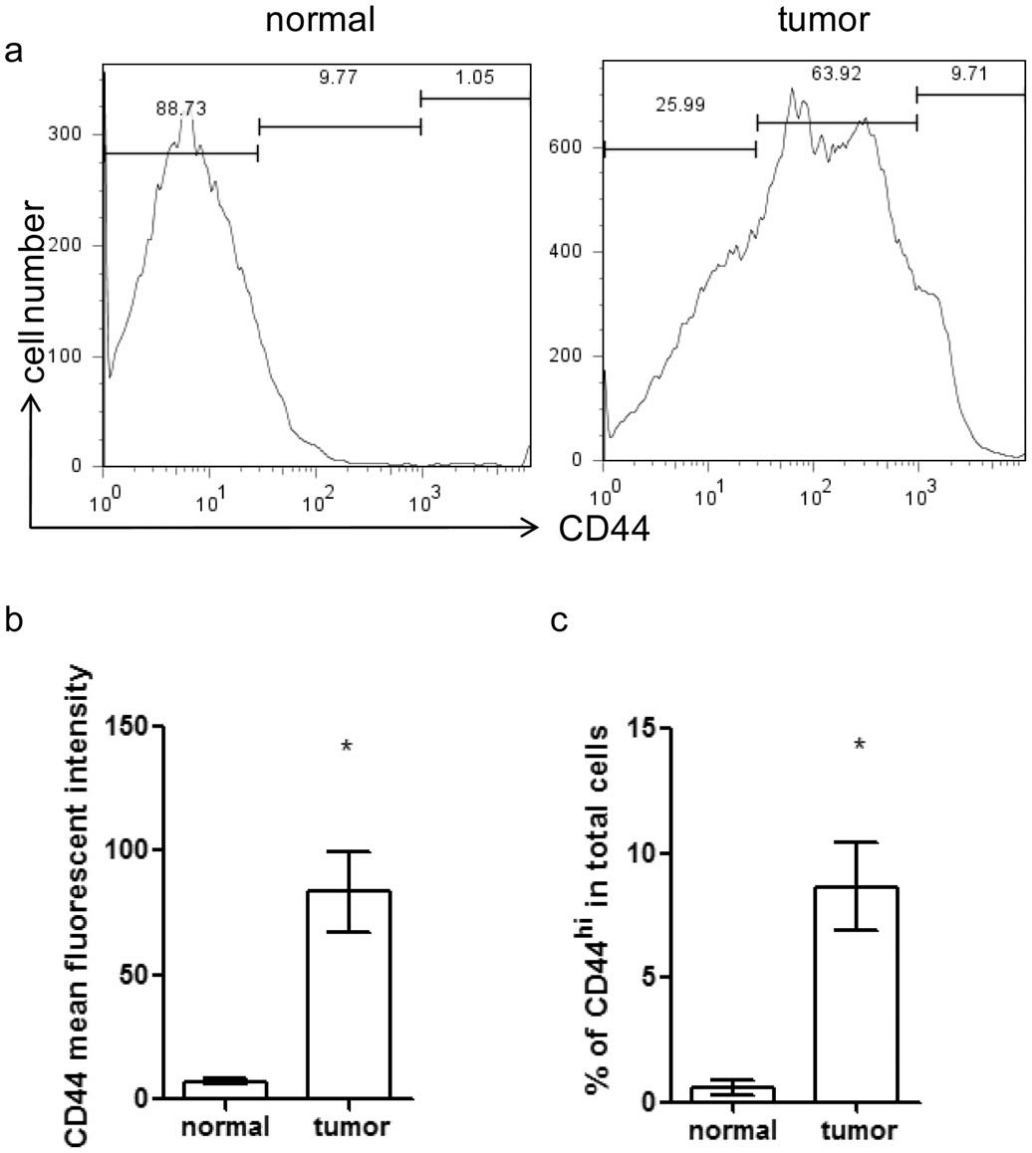
Frequency of CD44^hi^ cells in mouse salivary gland tumors. (a) Histograms of CD44 staining in normal salivary gland cells from wildtype mice (left panel) or tumor salivary gland cells from Plag1 transgenic mice (right panel). Representative graphs from of each group (n = 7) are shown. (b) CD44 mean fluorescent intensity in normal salivary gland cells from wildtype mice (left panel) or tumor salivary gland cells from Plag1 transgenic mice. n = 9, * p<0.05 (normal, vs. tumor, t-test); (c) Percentage of CD44^hi^ cells among total salivary gland cells in normal glands or tumor glands. n = 9, * p<0.05 (normal, vs. tumor, t-test).

Both normal salivary gland and tumor salivary gland cells displayed CD44 staining via FACS analysis. The mean fluorescent intensities (MFI) for CD44 was significantly higher on tumor cells than on wildtype cells (84 vs. 7, p<0.05) (Figure 1a, b). We further characterized the CD44 cells into CD44^hi^, CD44^intermediate^ (CD44^int^) and CD44^neg^ subpopulation by MFI. We noted that the CD44^hi^ population registered MFI that was one log higher than that of the CD44^neg^ population. The percentage of CD44^hi^ cells in the total salivary gland tumor cell population was 8.6%. This was significantly higher than the percentage of CD44^hi^ cells in the wildtype salivary glands (0.6%) (Figure 1c). Thus, CD44 protein expression was significantly upregulated in salivary gland tumor cells.

### Phenotype of CD44^hi^ tumor cells

**Figure 2:**
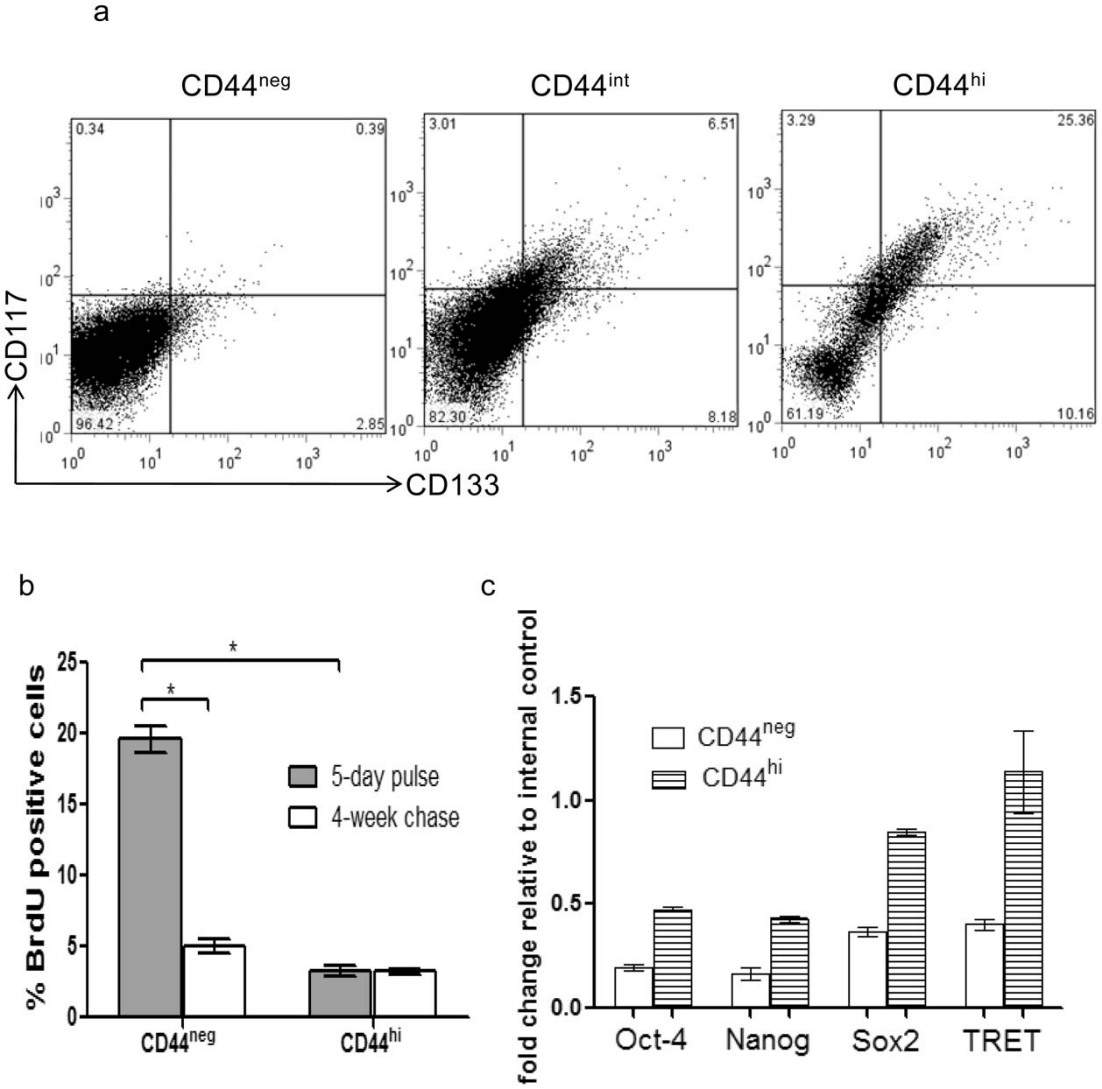
Phenotypic analysis of tumor cells. (a) CD117 and CD133 staining of tumor cells. Mice salivary gland tumor cells were divided into CD44^neg^, CD44^int^, and CD44^hi^ subpopulations via FACS; each subpopulation was analyzed for CD117 and CD133 expression. (b) BrdU incorporation. BrdU was injected into tumor-bearing mice at 50 mg/kg twice daily for 5 days, then half of the mice were sacrificed and half were rested for 4 weeks before CD44 and BrdU analysis. (n = 5 each group, * p<0.05 t-test). (c) Comparison of transcript levels for stem cell related genes in CD44^neg^ and CD44^hi^ tumor cells. Transcripts levels for all four genes were higher in CD44^hi^ cells than in CD44^neg^ cells (p<0.05, t-test).

CD133 and CD117 are two markers frequently associated with T-ICs [7], [10], [19]–[21]. We examined their expression in different CD44 subpopulations of tumor cells by flow cytometry. CD44^hi^ subpopulation contained cells which co-stained with CD117 and CD133 antibodies. These cells constituted about 25% of the total CD44^hi^ cells. In contrast, only 6% of CD44^int^ cells stained for CD117 and CD133, and less than 0.5% of the CD44^neg^ cells expressing CD117 and CD133 (Figure 2a).

Many studies have revealed that T-ICs do not divide as fast as their non-T-IC counterparts [22]–[24]. To determine if this was the case in salivary gland tumors, we compared the cell cycling rate and label retaining ability of CD44^hi^ and CD44^neg^ tumor cells (Figure 2b). After a 5-day pulse of BrdU, about 3.5% CD44^hi^ cells incorporated BrdU, compared to 19% of CD44^neg^ cells, indicating that CD44^hi^ cells were slow-cycling compared to CD44^neg^ cells. When the second group of mice was similarly pulsed with BrdU for 5 days, followed by a rest period of 4 weeks, about 3% CD44^hi^ cells were still BrdU positive, whereas only about 5% of CD44^neg^ cells retained BrdU marker. Therefore, majority of CD44^hi^ cells retained their BrdU over the course of 4 weeks whereas only a small fraction of CD44^neg^ cells were able to do so. The slow baseline cycling rate and robust BrdU retaining ability of CD44^hi^ cells were consistent with stem cell like properties [22]–[24].

Transcription factors such as Oct-4, Nanog, Sox2 and telomerase are implicated in linage commitment or maintenance, and function for cells of stem cell-like properties [25]–[29]. We performed realtime PCR to assess gene transcript levels for Oct4, Nanog, and Sox2, as well as mouse telomerase reverse transcriptase (mTRET), a catalytic subunit of telomerase, in CD44^hi^ and CD44^neg^ tumor cells. Transcript levels for those stem cell related factor were higher in CD44^hi^ cells than in CD44^neg^ cells (Figure 2c).

### High tumorigenicity of CD44^hi^ tumor cells *in vivo*

We have demonstrated with Plag1 transgenic mouse model that pleomorphic adenomas contained a population of CD44^hi^ cells that co-express other common T-ICs markers and propagate in slow-cycling state, and have upregulated stem cell related transcription factors. To determine if these CD44^hi^ cells could induce tumors in mice, *in vivo* tumorigenic experiments were performed. For all our *in vivo* transfer experiments, immunocompetent wildtype CD57/BL6 mice instead of immunocomprised NOD/SCID mice were used as hosts [30].

The pleomorphic adenoma tumor cells were sorted into various populations according to their surface markers. Comparable cell populations were injected into bilateral salivary glands of the same mouse. Unsorted 1×10^5^ tumor cells initiated tumors in about two thirds of the recipients, and 1×10^4^ unsorted tumor cells generated tumors in less than one third of the recipients. In contrast, as few as 1×10^3^ sorted CD44^hi^ tumor cells gave rise to tumors in a majority of recipients (7/9); 5×10^2^ sorted CD44^hi^ tumor cells generated tumors in one third of the recipients. For CD44^neg^ tumor cells, 1×10^5^ cells gave rise to tumors in more than half of recipients (5/9); 1×10^4^ tumor cells gave rise to tumor in one eighth of the recipients. CD44^hi^ cells showed 10–20 fold greater ability to initiate tumors in wildtype mice compared with the total number of tumor cells (Table 1), suggesting that the CD44^hi^ population was enriched for T-ICs.

**Table 1:** Tumorigenicity of tumor cell subpopulations in vivo.

	number of cells transferred					
cell type	1×10^6^	1×10^5^	1×10^4^	1×10^3^	5×10^2^	2×10^2^
**Unsorted cell**	8/8	6/9	2/9	0/9	0/9	0/9
**CD44^hi^**	7/7	9/9	8/8	7/9	3/9	0/9
**CD44^neg^**	8/8	5/9	1/8	0/9	0/9	0/9
**CD44^hi^CD133^+^CD117^+^**	4/4	5/5	8/8	9/9	6/9	3/9
**CD44^neg^CD133^+^CD117^+^**		3/7	2/8	1/9	0/9	0/9

Subpopulations of tumor cells were sorted with markers as indicated and transferred at indicated doses to wildtype mice recipients. Recipients were monitored up to 3 months after transfer for tumor formation. Unsorted tumor cells were used as control. (numbers in the table: tumor bearing mice/injected mice).

We next asked whether CD117 and CD133 can be used as T-ICs markers in our tumor model. When CD44^neg^CD117^+^CD133^+^ tumor cells were transplanted at the dose of 1×10^4^ cells, two out of eight recipients developed a secondary tumor. However, when CD44^hi^CD117^+^ CD133^+^ triple marker positive tumor cells were sorted and transplanted into the recipients, these cells initiated secondary tumors in one third of the recipients at the dose of 2×10^2^ cells. Thus, T-ICs of pleomorphic adenomas were further enriched in CD44^hi^CD117^+^CD133^+^ populations, but not CD44^neg^CD117^+^CD133^+^ populations.

**Figure 3:**
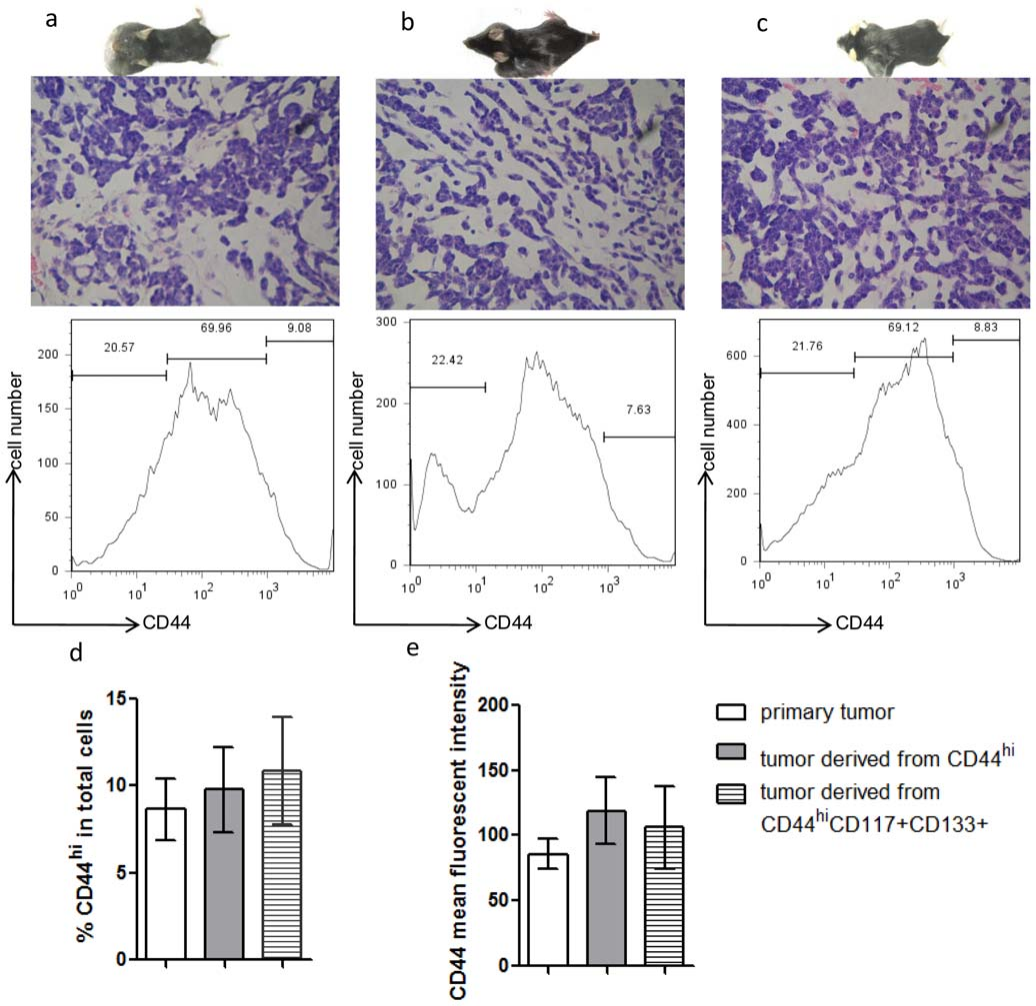
Histological and cell surface marker analysis for secondary tumors. (a–c) Each panel includes a picture of tumor bearing mouse (top), H&E staining (400×) of the tumor (middle), and tumor cell CD44 staining detected by FACS (bottom). (a) Primary tumors from Plag1 transgenic mice. Mice developed bilateral salivary gland tumors. (b) Tumors derived from CD44^hi^ tumor cell transplant. Mice were injected with 1×10^3^ CD44^hi^ cells at left side and 1×10^3^ CD44^neg^ cells at right side as control. Tumors only developed on the left side of the recipients. (c) Tumors derived from CD44^hi^CD117^+^CD133^+^ tumor cells transplant. Mice were injected with 1×10^3^CD44^hi^CD117^+^CD133^+^ cells at left side and 1×10^3^ CD44^neg^CD117^+^CD133^+^ cells at right side as control. Tumors only developed at left side of the recipients. (d) Percentage of CD44^hi^ cells among total cells in primary tumors (open bar), among total cells in tumors derived from CD44^hi^ cells (grey-color filled bar), and among total cells in tumors derived from CD44^hi^ CD117^+^CD133^+^ cells (shaded bar) (n = 9, P>0.05 between any two groups). (e) CD44 mean fluorescent intensity in primary tumors (open bar), in tumors derived from CD44^hi^ cells (grey-color filled bar), and in tumors derived from CD44^hi^ CD117^+^CD133^+^ cells (shaded bar) (n = 9, P>0.05 between any two groups).

Tumors initiated by CD44^hi^ or CD44^hi^CD117^+^CD133^+^ cells shared similar histological features with the primary tumors (Figure 3a–c). They often form luminal or cyst-like structures, and also contained myoepithelial cells in a disordered pattern. CD44 expression was similar (6–10%) in both the primary and secondary tumors initiated by CD44^hi^ or CD44^hi^CD117^+^CD133^+^ cells transplants (Figure 3a–d). Mean fluorescent intensity analysis of CD44 revealed that all three groups of tumors expressed similar levels of CD44 (Figure 3e). Secondary tumors initiated by CD44^hi^ or CD44^hi^CD117^+^CD133^+^cells predominantly contained CD44^neg^ cells. Therefore, CD44^hi^ tumor cells were able to produce secondary tumors that resemble primary tumors, consisting of both CD44^hi^ and CD44^neg^ cells.

### Plag1 upregulates Egr1 transcription

**Figure 4:**
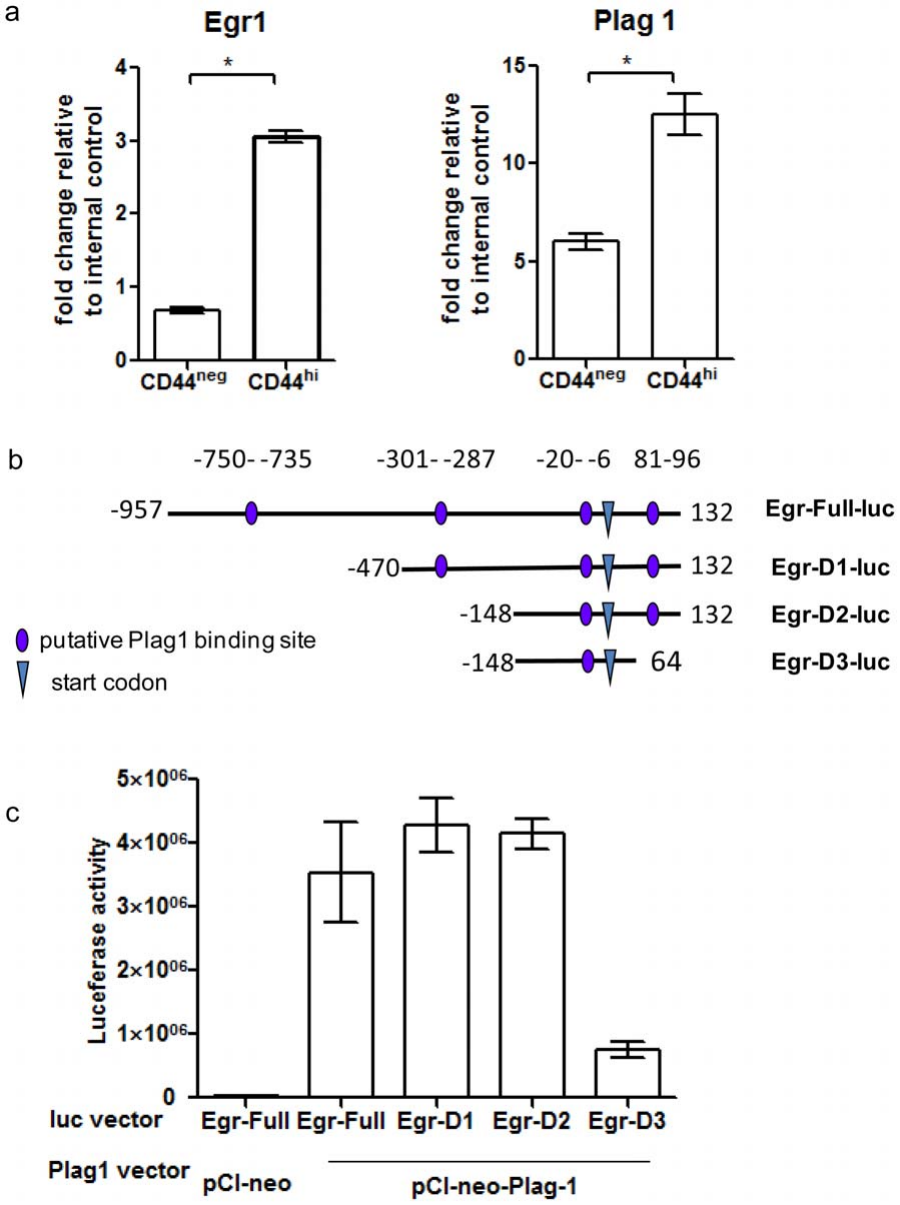
Plag1 upregulated Egr1 transcription in NIH3T3 cells. (a) Plag1 and Egr1 transcripts levels in CD44^hi^ and CD44^neg^ tumor cells were determined by real-time PCR. Expression levels were normalized to Ppib gene as internal control (* p<0.05). (b) Schematic diagram of Egr1 promoter region and truncated forms used. Four putative Plag1 binding sequences are marked in the mouse Egr1 promoter region −750 to 132. Egr-Full-luc contains all four sequences, Egr-D1-luc lacked binding sequence in region −750–−735, Egr-D2-luc lacked binding sequence in region −301–−287, Egr-D3-luc lacked binding sequence in region 81–96. (c) Transactivation of Egr1 promoter by Plag1. Co-transfection of vector pCI-neo-Plag1 and luciferase reporter driven by truncated forms of Egr1 promoter in NIH3T3 cells. As expected, pCI-neo empty vector co-transfected with Full-luc did not induce significant luciferase activity. Relative luciferase activity was normalized with β-galactosidase activity derived from pSV-β-galactosidase control vector. Results were shown as average of triplicates.

Egr1, Early Growth Response 1, a positive regulator of CD44 [31]–[32], was found to be significantly upregulated in Plag1 transgenic mice tumors (unpublished microarray analysis). Plag1 and Egr1 gene transcript levels were assessed by realtime quantitative PCR with cDNA extracted from CD44^hi^ and CD44^neg^ tumor cells. Both Plag1 transcripts and Egr1 transcripts levels were significantly higher in CD44^hi^ cells than in CD44^neg^ cells (Figure 4a).

Egr1 promoter sequence analysis revealed 4 putative Plag1 binding consensus sites (Figure 4b). Co-transfection of Plag1 expression vector (pCI-neo-Plag1) and mouse Egr1 reporter construct containing all four binding sequences induced robust luciferase activity. This data indicated that Plag1 could serve as a positive regulator for Egr1 transcription. To identify the promoter region important for Plag1-mediated upregulation of Egr1, truncated forms of Egr1 promoters were constructed to contain only 3 out of 4 putative binding sequences (Figure 4b). Deleting the binding sequences in Egr1 promoter region −750–−735 or −301–−287 did not dampen the effects of Plag1 on Egr1 upregulation. However, when the Egr1 promoter sequence in region 81–96 was deleted, upregulation of Egr1 induced by Plag1 was almost completely abolished (Figure 4c). These results demonstrated that promoter sequences 81–96 were necessary for Plag1-mediated Egr1 upregulation.

## Discussion

A majority of T-IC studies employ human tumor xenotransplants that utilize immunocompromised mice without a functional adaptive immune system (T and B lymphocytes) as the recipients of human T-ICs [33]. However, these mice still harbor a functioning innate immune system which could cause cellular ‘graft’ rejection. Immune rejection of transplanted T-ICs would result in an inaccurate assessment of the frequency of T-ICs in tumors [30], [34]. A recent study by Quintana et al. highlighted the impact of immune rejection on T-IC frequency estimations [29]. Using a NOD/SCID mice model with common gamma chains knocked out (NOD/SCID IL2g*^−/−^*), they showed that human melanoma cells initiated tumors 100% of the time upon transplantation into NOD/SCID IL2g*^−/−^* hosts. However, with conventional NOD/SCID recipients, the frequency of tumor-initiating cells was found to be about 0.1% [34]. Intrinsic differences among various immunodeficient mouse strains could account for the T-IC frequency variability, and such discrepancies could greatly confound the interpretation of experimental data. Therefore, assessing the efficacy of T-IC models in syngeneic transplantation systems is a critical and imperative step that formed the foundation and basis of our testing T-ICs in syngeneic, immunocompetent hosts.

We found that salivary gland tumors induced by Plag1 overexpression contained CD44^hi^ cells that incorporated BrdU at a low cycle rate and retained BrdU better than CD44^neg^ cells; and that CD44^hi^ cells have higher levels of transcripts of stem cell related genes, Oct-4, Nanog, Sox2 and telomerase. These data collectively suggest a stem cell-like phenotype for CD44^hi^ tumor cells. A few hundred transplanted tumor cells were able to initiate new tumors in syngeneic recipients. Secondary tumors shared the same histopathological features and cellular composition as their parent tumors. These findings supported previous reports that identified CD44 as a stem cell marker in colon, prostate cancer, breast, and head and neck squamous cell carcinomas [22], [35]–[37]. These studies and our results (Figure 1) indicated that CD44 expression was widely distributed across a number of tumors and a range of CD44 expression levels—negative to very high levels—were observed in the tumor cells. This broad pattern of CD44 expression across various tumor types could lead to inaccurate analyses of CD44^+^ tumor populations. Hence, Chu et al. categorized tumor cells into CD44^hi^ and CD44^neg^ populations [22], where CD44^hi^ populations were defined by cells exhibiting a mean fluorescent intensity (MFI) of at least half a log higher than CD44^neg^ cells. In this study, we defined CD44^hi^ cells as exhibiting MFI that was one log higher than that of CD44^neg^ cells. This is a more stringent criterion. Our results revealed that in murine salivary gland tumor, CD44^hi^ population was enriched for T-ICs as well.

CD113 is a marker expressed on hematopoietic stem cells isolated from cord blood. The use of CD133 as a T-IC marker is still debatable, largely due to the variability in its tissue-specific expressions. For example, in a colon adenocarcinoma xenotransplant model, CD133^+^ cells did not display increased tumorigenicity [22] even though they have been reportedly found in T-ICs [38]. The intrinsic difference among these tumor models could contribute to this discrepancy. CD117, a cytokine receptor expressed on hematopoietic stem cells and other cell types, has been detected on the surfaces of various tumors stem cells, including those found in leukemia and lung cancer [20]–[21]. Our study showed higher CD133 and CD117 expression levels in tandem with CD44^hi^ cells, when compared with the CD44^neg^ population. Triple marker CD44^hi^CD133^+^ CD117^+^ cells possessed potent tumor initiating capacities, indicating that CD44^hi^ CD133^+^ CD117^+^ could be used as a robust marker for T-IC identification in salivary gland tumors.

Although CD44^+^ markers have been widely used to isolate and identify T-ICs, it was not until recent that the functional roles of CD44 in T-ICs were investigated [11]. Du et al. used RNA interference to knockdown CD44 and CD133 in colorectal T-ICs isolated from patients. They showed that the deletion of CD44, and not CD133, strongly prevented clonal formation and inhibited the tumorigenicity of T-ICs in a xenografted model [11]. Therefore, the authors deduced that CD44 not only functioned as a marker for T-ICs, it could also participate in tumorigenic signaling pathways.

Our data showed that both Plag1and Egr1 gene transcriptions were upregulated in murine salivary gland CD44^hi^ tumor cells compared to CD44^neg^ cells (Figure 4a). We also demonstrated that Plag1 activated Egr1 gene transcription, and Egr1 promoter region 81–96 was necessary in mediating this process (Figure 4c). The CD44 gene contains an Egr1 binding site at −301 in the promoter region, and this Egr1 binding motif is indispensable to the stimulus-induced expression of CD44 [31]–[32]. These data collectively indicate a plausible Plag1-Egr1-CD44 regulatory pathway in the T-ICs of pleomorphic adenomas.

### Conclusion

Our study demonstrated that CD44^hi^ cells function as the T-ICs in pleomorphic adenomas that are capable of generating secondary tumors with similar histopathological features to the parent tumors. In addition, we provided evidence that Plag1 was capable of activating gene transcription of Egr1, an established upregulator for CD44, suggesting that Plag1 may be potentially involved in upregulation of CD44.

## Materials and Methods

### MMTV-PLAG1 transgenic mice

Mouse mammary tumor virus (MMTV)-PLAG1 transgenic mice, maintained in C57BL/6 background were generated at Experimental Animal Center, Department of Medical Genetics, Shanghai Jiaotong University School of Medicine [17]–[18]. All transgenic mice used in this study were genotyped via PCR, and were all heterozygous for the Plag1 transgene. Almost all the transgenic mice developed submandibular gland pleomorphic adenomas between 4–12 weeks old. Age (6–8 weeks old) and sex-matched C57BL/6 wildtype mice were used as controls. All the procedures described were approved by the Animal Use and Care Committee of Shanghai Jiaotong University School of Medicine (approval number: SYKX-2008-0050).

### Preparation of single cell suspensions from salivary gland tumors and normal salivary gland tissues

To generate single cell suspensions from salivary gland tumors, tumor samples from Plag1 transgenic mice were minced with scissors, mixed with 0.25% trypsin-EDTA and incubated at 37°C for 1 hour in a shaking water bath. Following incubation, cells were collected after blocking with PBS containing 10% FBS (Gibco, Carlsbad, CA, USA) and serially filtered through 200 and 100 µm nylon meshes. Contaminated red blood cells were removed by osmotic lysis and the filtered cells were suspended in PBS/1%FBS. Similarly, normal salivary gland tissues from C57BL/6 wildtype mice were also prepared into single cell suspensions.

### Flow cytometry

The antibodies used in the study included immunoglobulins, anti-CD44 allophycocyanin (APC), anti-CD133 phycoerythrin (PE), anti-CD117 fluorescein isothiocyanate (FITC) and anti-BrdU (FITC) (eBioscience, San Diego, CA, USA). Via-probe 7- Amino-actinomycin D (BD, San Jose, CA, USA) was used to gate live cells.

For cell surface staining, cells were counted and transferred to a 15-ml tube before they were washed and re-suspended. To minimize the unspecific binding of antibodies, cells were first incubated with mouse immunoglobulins at 0.06–0.1 µg/1×10^6^ cells on ice for 10 minutes, after which the samples were washed twice. Fluorescent-conjugated antibodies were then mixed with the re-suspended cells, incubated for another 30 minutes on ice and washed twice again.

To label the cells and investigate their BrdU retaining abilities, the mice were injected with BrdU at 50 mg/kg twice daily for 5 days. They were then divided into two groups, where one was sacrificed immediately and the other group was left alone for 4 weeks. Cell surface staining was then performed on the single cell suspensions before being permeablized with 1% PFA and 0.5% Tween-20 in PBS for 30 minutes at room temperature. This was followed by DNaseI treatment at 100 U/ml (Sigma, St Louis, MO, USA) in a 37°C water bath for 15 minutes. Anti-BrdU antibodies were then added into cells for 45 minutes at room temperature. FACS Caliber (BD, San Jose, CA, USA) was used for cell acquisition and CellQuest (BD, San Jose, CA, USA) was used for analysis. For all analyses, live cells were gated based on 7-AAD negative staining.

### Cell sorting and tumorigenicity experiments

Cell sorting was performed using a BD FACSAria cell sorter. Sorted cells were spun down by low speed centrifugation(1000 rpm for 5 min) and re-suspended in RPMI 1640 supplemented with 10% FBS, 20 mM Hepes and 2 mM L-glutamine. In all the experiments, a small aliquot of cells was set aside to confirm cell counts and viability. Once confirmed, cells were diluted to appropriate injection doses and injected into murine submandibular glands with 28G needles and microliter syringes (Hamilton, Reno, NV, USA) under anesthesia. To minimize experimental variability, comparative groups of cells were injected into the contralateral submandibular glands of the same animal. The injected mice were followed up intensively for the subsequent 3 months.

### Realtime PCR

RNA from salivary gland tissues and tumor tissues were extracted with TRIzol (Invitrogen, Carlsbad, CA, USA) according to the manufacturer's instructions. RNA samples were subsequently treated with DNase I (Invitrogen, Carlsbad, CA, USA) twice to avoid DNA contamination, followed by reverse-transcription into cDNAs using AMV reverse transcriptase (TaKaRa, Shiga, Japan). Primers for realtime PCR on cDNA samples were: Plag1: GTTTCTAAGTACAAATTAC; CATGTGTATGGAGGTGATT; Egr1: GAGCGAACAACCCTATGAGC; AGGCAGAGGAAGACGATGAA; Oct4: GAGGAGTCCCAGGACATGAA; TGGTCTCCAGACTCCACCTC; Nanog: AAGTACCTCAGCCTCCAGCA; GCTTGCACTTCATCCTTTGG; Sox2: ATGATGGAGACGGAGCTGAA; TTGCTGATCTCCGAGTTGTG; mTERT: GGATTGCCACTGGCTCCG; TGCCTGACCTCCTCTTGTGAC; Primers for PPib (internal control) include: TCGTCTTTGGACTCTTTGGAA; TCCTTGATGACACGATGGAA.

### Transfection and reporter gene assays

NIH3T3 cells were maintained in Dulbecco's modified Eagle's medium supplemented with 10% fetal calf serum (Gibco, Carlsbad, CA, USA), 50 U/mL streptomycin and 4 mM l-glutamine. The NIH3T3 cells were then transfected with plasmid DNA using FuGENE 6 (Roche, Indianapolis, IN, USA). Mouse (−957 to 132) Egr1 promoter fragments were cloned into pGL3-basic (Promega, Madison, Wisconsin, USA) and named Egr-Full-luc. For deletional studies, Egr1 promoter fragment −470–132 was cloned into pGL3-basic and named Egr-D1-luc; fragment −148–132 was cloned into pGL3-basic and named Egr-D2-luc; fragment −148–64 was cloned into pGL3-basic and named Egr-D3-luc. Plag1 gene expression vector pCI-neo-Plag1 and empty pCI-neo vector were co-transfected into NIH3T3 cells in triplicate on 48-well plates with an Egr-luc reporter construct (Egr-Full-luc or Egr-D1-luc or Egr-D2-luc or Egr-D3-luc). Luciferase activity was measured 48 hr after transfection with luciferase assay system (Promega, Madison, Wisconsin, USA) according to the manufacturer's instruction. Relative luciferase activity was normalized with β-galactosidase activity derived from pSV-β-galactosidase control vector (Promega, Madison, Wisconsin, USA).

## References

[1] Reya T, Morrison SJ, Clarke MF, Weissman IL (2001). Stem cells, cancer, and cancer stem cells.. Nature.

[2] Chan KS, Espinosa I, Chao M, Wong D, Ailles L (2009). Identification, molecular characterization, clinical prognosis, and therapeutic targeting of human bladder tumor-initiating cells.. Proc Natl Acad Sci U S A.

[3] Dubrovska A, Kim S, Salamone RJ, Walker JR, Maira SM (2009). The role of PTEN/Akt/PI3K signaling in the maintenance and viability of prostate cancer stem-like cell populations.. Proc Natl Acad Sci U S A.

[4] Hong SP, Wen J, Bang S, Park S, Song SY (2009). CD44-positive cells are responsible for gemcitabine resistance in pancreatic cancer cells.. Int J Cancer.

[5] Bonnet D, Dick JE (1997). Human acute myeloid leukemia is organized as a hierarchy that originates from a primitive hematopoietic cell.. Nat Med.

[6] Fillmore C, Kuperwasser C (2007). Human breast cancer stem cell markers CD44 and CD24: enriching for cells with functional properties in mice or in man?. Breast Cancer Res.

[7] Wright MH, Calcagno AM, Salcido CD, Carlson MD, Ambudkar SV (2008). Brca1 breast tumors contain distinct CD44^+^/CD24^−^ and CD133^+^ cells with cancer stem cell characteristics.. Breast Cancer Res.

[8] Dontu G, Al-Hajj M, Abdallah WM, Clarke MF, Wicha MS (2003). Stem cells in normal breast development and breast cancer.. Cell Prolif.

[9] Pallini R, Ricci-Vitiani L, Banna GL, Signore M, Lombardi D (2008). Cancer stem cell analysis and clinical outcome in patients with glioblastoma multiforme.. Clin Cancer Res.

[10] McCord AM, Jamal M, Williams ES, Camphausen K, Tofilon PJ (2009). CD133^+^ glioblastoma stem-like cells are radiosensitive with a defective DNA damage response compared with established cell lines.. Clin Cancer Res.

[11] Du L, Wang H, He L, Zhang J, Ni B (2008). CD44 is of functional importance for colorectal cancer stem cells.. Clin Cancer Res.

[12] Singh SK, Hawkins C, Clarke ID, Squire JA, Bayani J (2004). Identification of human brain tumour initiating cells.. Nature.

[13] Dalerba P, Dylla SJ, Park IK, Liu R, Wang X (2007). Phenotypic characterization of human colorectal cancer stem cells.. Proc Natl Acad Sci U S A.

[14] Laskawi R, Schott T, Schroder M (1998). Recurrent pleomorphic adenomas of the parotid gland: clinical evaluation and long-term follow-up.. Br J Oral Maxillofac Surg.

[15] Sabesan T, Ramchandani PL, Hussein K (2007). Metastasising pleomorphic adenoma of the parotid gland.. Br J Oral Maxillofac Surg.

[16] Declercq J, Van Dyck F, Braem CV, Van Valckenborgh IC, Voz M (2005). Salivary gland tumors in transgenic mice with targeted PLAG1 proto-oncogene overexpression.. Cancer Res.

[17] Zhao X, Ren W, Yang W, Wang Y, Kong H (2006). Wnt pathway is involved in pleomorphic adenomas induced by overexpression of PLAG1 in transgenic mice.. Int J Cancer.

[18] Zhao XD, Yang WJ, Wang L, Kong H, Ren WH (2003). [Development of salivary gland tumors in pleomorphic adenoma gene 1 transgenic mice].. Zhonghua Yi Xue Yi Chuan Xue Za Zhi.

[19] Zhu Z, Hao X, Yan M, Yao M, Ge C (2010). Cancer stem/progenitor cells are highly enriched in CD133(+)CD44(+) population in hepatocellular carcinoma.. Int J Cancer.

[20] Levina V, Marrangoni A, Wang T, Parikh S, Su Y (2010). Elimination of human lung cancer stem cells through targeting of the stem cell factor-c-kit autocrine signaling loop.. Cancer Res.

[21] Guibal FC, Alberich-Jorda M, Hirai H, Ebralidze A, Levantini E (2009). Identification of a myeloid committed progenitor as the cancer-initiating cell in acute promyelocytic leukemia.. Blood.

[22] Chu P, Clanton DJ, Snipas TS, Lee J, Mitchell E (2009). Characterization of a subpopulation of colon cancer cells with stem cell-like properties.. Int J Cancer.

[23] Taylor G, Lehrer MS, Jensen PJ, Sun TT, Lavker RM (2000). Involvement of follicular stem cells in forming not only the follicle but also the epidermis.. Cell.

[24] Al-Hajj M, Clarke MF (2004). Self-renewal and solid tumor stem cells.. Oncogene.

[25] Yu J, Vodyanik MA, Smuga-Otto K, Antosiewicz-Bourget J, Frane JL (2007). Induced pluripotent stem cell lines derived from human somatic cells.. Science.

[26] Takahashi K, Tanabe K, Ohnuki M, Narita M, Ichisaka T (2007). Induction of pluripotent stem cells from adult human fibroblasts by defined factors.. Cell.

[27] Lin T, Meng L, Li Y, Tsai RY (2010). Tumor-initiating function of nucleostemin-enriched mammary tumor cells.. Cancer Res.

[28] Smith BH, Gazda LS, Conn BL, Jain K, Asina S (2011). Three-dimensional culture of mouse renal carcinoma cells in agarose macrobeads selects for a subpopulation of cells with cancer stem cell or cancer progenitor properties.. Cancer Res.

[29] Gillis AJ, Stoop H, Biermann K, van Gurp RJ, Swartzman E, Cribbes S (2011). Expression and interdependencies of pluripotency factors LIN28, OCT3/4, NANOG and SOX2 in human testicular germ cells and tumours of the testis.. Int J Androl.

[30] Dick JE (2009). Looking ahead in cancer stem cell research.. Nat Biotechnol.

[31] Fitzgerald KA, O'Neill LA (1999). Characterization of CD44 induction by IL-1: a critical role for Egr-1.. J Immunol.

[32] Maltzman JS, Carman JA, Monroe JG (1996). Role of EGR1 in regulation of stimulus-dependent CD44 transcription in B lymphocytes.. Mol Cell Biol.

[33] Dalerba P, Cho RW, Clarke MF (2007). Cancer stem cells: models and concepts.. Annu Rev Med.

[34] Quintana E, Shackleton M, Sabel MS, Fullen DR, Johnson TM (2008). Efficient tumour formation by single human melanoma cells.. Nature.

[35] Al-Hajj M, Wicha MS, Benito-Hernandez A, Morrison SJ, Clarke MF (2003). Prospective identification of tumorigenic breast cancer cells.. Proc Natl Acad Sci U S A.

[36] Okamoto A, Chikamatsu K, Sakakura K, Hatsushika K, Takahashi G (2009). Expansion and characterization of cancer stem-like cells in squamous cell carcinoma of the head and neck.. Oral Oncol.

[37] Patrawala L, Calhoun T, Schneider-Broussard R, Li H, Bhatia B (2006). Highly purified CD44^+^ prostate cancer cells from xenograft human tumors are enriched in tumorigenic and metastatic progenitor cells.. Oncogene.

[38] Ricci-Vitiani L, Lombardi DG, Pilozzi E, Biffoni M, Todaro M (2007). Identification and expansion of human colon-cancer-initiating cells.. Nature.

